# Cystic Fibrosis, CFTR, and Colorectal Cancer

**DOI:** 10.3390/ijms21082891

**Published:** 2020-04-21

**Authors:** Patricia Scott, Kyle Anderson, Mekhla Singhania, Robert Cormier

**Affiliations:** Department of Biomedical Sciences, University of Minnesota Medical School, Duluth, MN 55812, USA; pscott@d.umn.edu (P.S.); and03632@umn.edu (K.A.); singh724@d.umn.edu (M.S.)

**Keywords:** CFTR, cystic fibrosis, colorectal cancer, tumor suppressor

## Abstract

Cystic fibrosis (CF), caused by biallelic inactivating mutations in the *cystic fibrosis transmembrane conductance regulator* (*CFTR*) gene, has recently been categorized as a familial colorectal cancer (CRC) syndrome. CF patients are highly susceptible to early, aggressive colorectal tumor development. Endoscopic screening studies have revealed that by the age of forty 50% of CF patients will develop adenomas, with 25% developing aggressive advanced adenomas, some of which will have already advanced to adenocarcinomas. This enhanced risk has led to new CF colorectal cancer screening recommendations, lowering the initiation of endoscopic screening to age forty in CF patients, and to age thirty in organ transplant recipients. The enhanced risk for CRC also extends to the millions of people (more than 10 million in the US) who are heterozygous carriers of *CFTR* gene mutations. Further, lowered expression of *CFTR* is reported in sporadic CRC, where downregulation of *CFTR* is associated with poor survival. Mechanisms underlying the actions of *CFTR* as a tumor suppressor are not clearly understood. Dysregulation of Wnt/β-catenin signaling and disruption of intestinal stem cell homeostasis and intestinal barrier integrity, as well as intestinal dysbiosis, immune cell infiltration, stress responses, and intestinal inflammation have all been reported in human CF patients and in animal models. Notably, the development of new drug modalities to treat non-gastrointestinal pathologies in CF patients, especially pulmonary disease, offers hope that these drugs could be repurposed for gastrointestinal cancers.

## 1. Overview

In 2020, worldwide, it is estimated that there will be more than 1.8 million new cases of colorectal cancer (CRC) and close to 900,000 deaths, with more than 50,000 CRC deaths in the US [1]. Overall, CRC is the major site of cancer incidence and mortality in the digestive system. Genetic susceptibility to CRC is considered high. While only ~5% of CRC is inherited in a dominant fashion (e.g., Lynch syndrome and familial adenomatous polyposis), it has been proposed that between 25% and 50% of CRCs demonstrate some form of familial susceptibility [2]. Notably, the full constellation of driver genes for colorectal cancer, especially their contributions to genetic disease susceptibility and their underlying mechanisms of action remain to be fully elucidated. One of the most recently described familial CRC syndromes involves individuals with cystic fibrosis (CF) caused by biallelic inactivating mutations in the *cystic fibrosis transmembrane conductance regulator* (*CFTR*) gene, who are highly susceptible to early, aggressive colorectal tumor development [3]. The enhanced risk for CRC also extends to heterozygous carriers of *CFTR* mutations [4]. Further, low expression of *CFTR* is associated with poor survival in sporadic CRC [5]. Thus, CFTR deficiency may impact on CRC risk and mortality in large segments of the population. This review focuses on the important role of the CFTR ion channel in colorectal cancer in individuals with germline *CFTR* mutations (both CF patients and carriers) and in sporadic cases of CRC.

### 1.1. Cystic Fibrosis

Biallelic inactivating germline mutations in the *CFTR* gene found on chromosome 7 cause the inherited life-threatening disease cystic fibrosis, the most common autosomal recessive disease among people of European (primarily northern Europe) ancestry [6]. *CFTR* produces an mRNA transcript of 6128 nucleotides [7] encoding a protein of 1480 amino acids that functions as a chloride (Cl^−^) and bicarbonate (HCO3^−^) anion channel. CFTR is located on the apical surfaces of luminal epithelia. While the most severe clinical manifestations of CF are found in the lung [8], *CFTR* is expressed in a variety of extra-pulmonary tissues [9,10,11], where its deficiency is associated with CF disease pathologies. Intestinal CFTR-deficiency causes obstruction in the ileum (infantile meconium ileus) and proximal colon [11]. CF causes nutrient malabsorption linked to pancreatic enzyme deficiency and potentially, defective uptake of lipids [11]. CF patients are also susceptible to intestinal celiac disease, which results from aberrant T_H_1 immune and antibody responses caused by eating gluten, a protein found in wheat, barley, and rye [12]. 

### 1.2. CFTR: Its Role in Intestinal Tract Homeostasis

Chloride channels, including CFTR, play a key role in homeostasis of the gastrointestinal (GI) tract. Their functions involve osmoregulation, transport of major ions across epithelia, polarity of cells, cellular metabolism including glucose metabolism, cellular autophagy and protein turnover, migration of cells, mucus secretion, innate and adaptive immune responses, cell–cell interactions, membrane potential, mitochondrial function and related oxidative stress, tissue inflammation, microbiota composition, cellular pH, programmed cell death, and intestinal stem cell regulation [13]. Notably, all of these functions are linked to the well-characterized hallmarks of cancer. As the GI tract is constantly exposed to environmental insults dysregulation of these ion channel functions can readily contribute to carcinogenesis [13]. 

CFTR is expressed along the entire length of the intestinal tract, with a gradient of decreasing expression proximal (duodenum) to distal (ileum) in the small intestine. In both the small and large intestine CFTR expression is strongest at the base of the crypt, the location of the intestinal stem cell compartment [14,15]. Some CFTR expression is also located on the brush border of villus cells, and there are rare CFTR-high-expressing cells scattered along the small intestine outside of the crypt base [16]. CFTR expression in the colon follows an expression gradient of highest in the cecum and proximal colon to lower amounts in the distal colon. The membrane-spanning domains of CFTR form an aqueous channel that permits the passage of Cl^−^ and HCO_3_^−^ ions down their electrochemical gradients, which in the intestine is from the cytoplasm of epithelial cells to the intestinal lumen, especially the intestinal crypt lumen. This movement of ions out of the cell increases osmotic pressure for the passage of water in the same direction. Thus, indirectly, CFTR also determines water homeostasis [17,18]. CFTR also regulates Na^+^, K^+^, Ca^2+^, and other Cl^−^ channels. For example, CFTR is implicated in inhibition of the activity of SCNN1 (sodium channel epithelial 1 alpha subunit), a Na^+^ channel, resulting in a further enhancement of water outflow in intestinal cells [19]. Further, CFTR is also implicated in maintenance of intestinal epithelial tight junctions, and in adjustments to the pH of cellular secretions. CFTR is also involved in sphingosine-1 phosphate (S1P) extracellular transport. S1P is a bioactive lipid mediator that is a critical regulator of inflammatory signaling and cell adhesion, among diverse roles [20]. In addition to its membrane-spanning domains, CFTR contains a cytoplasmic C-terminal PDZ (post-synaptic density protein 95 (PSD-95)-*Drosophila* discs large tumor suppressor (DLG1)-zona occludins 1 (ZO-1)-binding motif that interacts with PDZ-containing proteins that contribute to the regulation of intracellular signaling and the actin cytoskeleton [21,22]. Summarizing, in the GI tract, CFTR function is critical for ion and water homeostasis. Moreover, CFTR localization to the intestinal crypt stem cell compartment permits it to influence stem cell function and therefore, ultimately, CRC development as the intestinal stem cell is the likely cancer progenitor cell [23].

### 1.3. CF Patients Are at a High Risk for CRC

Development of more-effective therapeutic modalities for pulmonary dysfunction in people with CF has dramatically increased lifespan such that the expected average life expectancy of newborns with CF is ~44 years [24]. As people with CF have aged longer they are at increased risk for developing specific cancers. The first evidence for this was a 20-year epidemiological study using data from the US CF Registry comparing the incidence of cancer in people with CF to the predicted age-adjusted risk in the general population. It was reported that the risk of all types of GI cancers was increased and in particular the risk of CRC was increased 6-fold [25]. The same study also reported an enhanced risk for cancers of the esophago-gastric junction, biliary tract, small intestine, and testes, as well as for lymphoid leukemia. Further, meta-analysis of six population-based studies involving more than 99,000 patients confirmed the particular high risk for gastrointestinal cancers [26]. More directly, based on endoscopic screening studies, larger, more aggressive colon polyps were found in people with CF compared with non-CF individuals. These studies reported that by age forty 50% of individuals with CF developed adenomas. Moreover, half of these tumors were classified as aggressive advanced adenomas. Further, by age forty 3% of individuals with CF had already developed adenocarcinomas [3,27]. CF has now been declared a hereditary colon cancer syndrome by the Cystic Fibrosis Foundation [28]. It is now recommended that CRC endoscopic screening be initiated in CF patients by age forty, and in immunocompromised CF lung transplant patients, at particular risk for cancer, by age thirty [28].

### 1.4. CFTR Carriers Are Also Susceptible to GI Cancers

Germline *CFTR* mutations may be an enhanced risk factor for the estimated 3%–4% of the US population (>10 million people) that are heterozygote carriers for inactivating *CFTR* mutations [29] and therefore potentially at risk due to loss of heterozygosity (LOH) or haploinsufficiency. A recent population-based study using diagnostic codes to compare the prevalence of CF-related conditions in more than 19,000 *CFTR* heterozygous mutant carriers and 99,000 healthy controls, found that CF carriers were at an increased risk for 59 CF-related diagnostic conditions [4]. Specifically, carriers had a 44% increased prevalence of GI cancers (colon, stomach, and other GI organs). This increase was detected in spite of the fact that 96% of this cohort was under the age of 46 years [4] and thus this population was not representative of the age range in which most sporadic GI cancers occur, e.g., in the USA the average age for sporadic CRC is 68 years in males and 72 years in females, with a similar average age for gastric cancer [1].

### 1.5. CFTR Deficiency Causes Enhanced Risk in Sporadic CRC

*CFTR* deficiency is also linked to an increased risk for sporadic CRC. Than et al. reported that in a study of 90 Stage II CRC patients classified by tumor *CFTR* expression, 3-year disease-free survival (DFS) in the quartile of patients with lowest *CFTR* expression was 30% lower than in the remaining patients with higher CFTR expression [5]. A separate study in a different patient population by Sun et al. reported that *CFTR* mRNA and protein expression was lower in CRC tumors vs. normal tissue and CFTR mRNA expression was lower in metastatic CRC vs. non-metastatic CRC. Further, the same group found that *CFTR-*depleted CRC cell lines demonstrated more aggressive oncogenic characteristics including increased invasion, migration, and colony formation [30]. The factors that cause downregulation of CFTR expression in CRC are not known. However, intestinal-specific transcriptional regulatory elements have been identified. Enhancer elements in introns 1 and 11 have been shown to promote transcription [31] while the minor alleles of several exon 11 SNPs originally identified in CF patients caused decreased expression of CFTR in human Caco-2 CRC cell reporter assays [32]. Low CFTR expression in sporadic tumors may also be caused by silencing of the *CFTR* gene by promoter hypermethylation as was reported in other cancers such as lung, breast, head and neck, and bladder cancers [33,34,35,36,37,38].

### 1.6. Risk for GI Cancer Caused by CFTR Deficiency Was Confirmed in Mouse Genetic Studies

In *Sleeping Beauty* (SB) transposon-mediated genetic screens *Cftr* was identified as a common insertion site (CIS) gene in mouse GI tumors and likely candidate tumor suppressor gene. *Cftr* was ranked in the top 10% to 50% of CIS CRC cancer-driver genes in screens in *adenomatous polyposis coli* (*Apc)* wild-type [39], *Apc*-deficient [40], and *transforming growth factor-beta* (*TGF beta)*-deficient [41] genetic backgrounds. In a functional test of *Cftr* deficiency, Than et al. reported that in mice carrying an intestinal specific *Cftr* conditional knockout allele crossed to the tumor-sensitized *Apc^Min^* mouse, mice deficient for *Cftr* (both heterozygotes and homozygotes) developed significantly more tumors than *Apc^Min^* mice that were wildtype for *Cftr*. Furthermore, invasive lesions were only observed in the *Cftr*-deficient cohort. Notably, *Cftr*-deficiency alone was sufficient to cause tumors in >60% of *Apc* wildtype mice by 10–12 months of age [5]. 

## 2. Potential Mechanisms of CFTR Tumor Suppressor Action

### 2.1. CFTR Influences the Intestinal Stem Cell Compartment

Long-lived multipotent intestinal stem cells located at the crypt base self-renew and repopulate crypts with specialized differentiated cells (e.g., enterocytes, secretory cells)—every 4–5 days [15]. There is substantial evidence that these intestinal stem cells are the primary source of CRC progenitor cells [23]. *CFTR* expression in the colon is strongest at the crypt base in the stem cell compartment [14], with some reports indicating that CFTR is expressed by the intestinal stem cells [42]. There is additional evidence that CFTR influences intestinal cell lineage differentiation of mouse embryonic stem (ES) cells. Further, Li et al. reported that *CFTR* F508del mutant mouse ES cells developed teratomas, and upregulated genes involved in proliferation, migration, and epithelial to mesenchymal transition (EMT) [43]. Accordingly, *CFTR* is well-placed to influence intestinal crypt epithelial cell renewal and the activity of CRC progenitor cells. Intestinal crypt structure and function can be modeled in vitro using three dimensional (3D) organoid cultures. Organoids can be created from single intestinal stem cells or isolated crypt bases containing stem cells. Organoids retain normal intestinal crypt structure, polarity, and physiological functions, including self-renewal and lineage differentiation into all of the differentiated cell types in the crypt [44]. *Cftr* expression and activity have been tested in organoids created from both humans and mice, and CFTR has been shown to retain its normal Cl^−^ and HCO_3_^−^ ion function in these organoids [45,46]. Importantly, in several studies, colon organoids have been shown to be valuable surrogates to measure the effectiveness of therapeutic drugs to treat CF patients with specific *CFTR* mutations [47,48,49]. Further, organoids created from CRC cells or pre-cancerous tissues recapitulate the oncogenic phenotypes of these tissues [50,51]. Organoid cultures have been used to test the effect of *CFTR* deficiency on oncogenicity. For example, it was reported that *Cftr* knockout (KO) mouse colon organoids developed a significant increase in clonogenicity [5]. Further, organoids created from mouse small intestine also showed enhanced proliferation along with the localization of *Cftr* to the l*eucine rich repeat containing G protein-coupled receptor 5-*positive (LGR5+) intestinal stem cell [42]. Overall, these results confirm a likely role for *Cftr* in influencing the CRC progenitor cells and their microenvironment.

### 2.2. CFTR Is Linked to Regulation of Wnt/β-catenin Signaling

**Figure 1:**
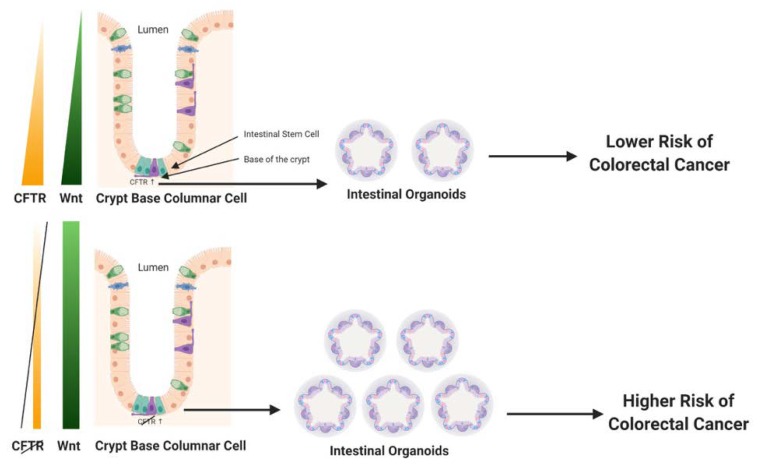
↓*Cystic fibrosis transmembrane conductance regulator* (CFTR) → ↑ Wnt/β-catenin signaling → ↑ proliferation. CFTR deficiency leads to increased cellular response to Wnt/β-catenin signaling and cellular proliferation. For example, CFTR deficiency enhances survival of colon organoids from CFTR knockout (KO) mice [5]. Increases in Wnt/β-catenin signaling is a hallmark of colorectal cancer (CRC) development. Figure created using BioRender.com.

Wnt/β-catenin signaling is a critical mediator of intestinal tissue homeostasis, in particular as a regulator of intestinal cell survival, proliferation, and differentiation, and key characteristics of normal intestinal cell renewal and function. Wnt/β-catenin expression and target signaling is tightly controlled. Wnt expression forms a gradient in the intestinal crypt, where its expression is strongest at the base of the crypt in the stem cell compartment (depicted in Figure 1, left panel). Notably, and not surprisingly, dysregulation of the Wnt/β-catenin signaling pathway is implicated in up to 90% of human CRC, strongly contributing to both early tumor initiation as well as promoting progression of invasive CRC [52]. Deficiency of *Cftr* in the mouse intestine has been linked to increased Wnt/β-catenin signaling. A study of intestinal tumors isolated from mice carrying an intestinal-specific conditional knockout of *Cftr* alone (*Apc* wildtype) found enhanced nuclear localization of β-catenin, an indicator of activation, along with elevated expression of Wnt/β-catenin target genes such as *Cyclin d1* (*Ccnd1*), *Lgr5*, and *cluster of differentiation 44* (*CD44*) [5]. This finding was confirmed in crypts and organoids from the small intestine of constitutive *Cftr* knockout mice. Compared with wildtype control mice, *Cftr* KO mice demonstrated enhanced proliferation, including a 30% increase in intestinal stem cell proliferation and a significant increase in Wnt/β-catenin signaling [42]. In the same study, deficiency for *Cftr* or a transient inhibition of channel activity by CFTR-(inh)-172 caused higher levels of intracellular pH in LGR5+ intestinal stem cells. This elevation in intracellular pH was linked to enhanced physical association of dishevelled segment polarity protein 2 (Dvl2), a key part of the Wnt/β-catenin signaling pathway, with phospholipids at the plasma membrane, thus placing Dvl2 in an ideal location to promote Wnt/β-catenin signaling [42]. However, it is noted that not all reports of *CFTR* deficiency in the intestine have found an upregulation of Wnt/β-catenin signaling. Liu et al. reported that in both F508del *Cftr* mutant mice and in the human Caco-2 CRC cell line deficient for CFTR there was decreased Wnt/β-catenin signaling. The authors of this study proposed that deficiency for CFTR physically disrupted β-catenin localization to the plasma membrane thus promoting its degradation in the proteasome, with this event eventually causing NF-κB (nuclear factor kappa-light-chain-enhancer of activated B cells) translocation to the nucleus leading to inflammation [53]. The significant differences between the Liu et al. study [53] and that of Than et al. [5] and Strubberg et al. [42] could be context dependent. For example, in the Liu et al. study total intestinal tissue was used for analysis, rather than intestinal epithelia, intestinal crypts, or organoids as was employed in Than et al. and Strubberg et al., thus the findings of Liu et al. could reflect an influence for *CFTR* independent of the stem cell compartment. Another contextual difference between the Liu et al. study and that of Than et al. and Strubberg et al. is in the specific mouse models of *Cftr* employed. Liu et al. employed the *Cftr*^tm1Kth^ mouse that mimics the F508del mutation. It is considered a hypomorph that retains some protein expression and that demonstrates 40% survival to maturity, even when untreated with a polyethelene glycol (PEG) osmotic laxative to prevent intestinal obstruction. The Liu et al. paper does not mention treatment of these animals with a PEG osmotic laxative, thus presumably there was no treatment. In contrast, Strubberg et al. (Cftr^tm1Unc^) and Than et al. (Cftr^fl10/fl10^ × intestinal Cre-mediated excision) employed more severe mutant *Cftr* alleles (as low as 5% survival to maturity) that required treatment of the mice with a PEG osmotic laxative (Colyte) to prevent lethality due to intestinal obstruction. Treatment with Colyte significantly reduces bacterial load and inflammation [54]. Accordingly, it is likely that the mice described in Liu et al. (depicted in Fig. 1 in that paper [53]) were undergoing a level of intestinal inflammation not observed in the Strubberg et al. and Than et al. studies where mice were treated with Colyte, and this difference could in part explain differences in gene expression reported in the respective papers. It is also noted that the influence of deficiency for *CFTR* on Wnt/β-catenin signaling was context-/tissue-dependent in other published studies by the Liu et al. research group [55]. Further, the role of Wnt/β-catenin signaling in CRC could vary by CRC stage. For example, while dysregulation of canonical Wnt/β-catenin signaling is widely considered to be the primary driver for early CRC development and progression [52], there are some reports that decreased Wnt/β-catenin target gene expression is implicated in poor prognosis for some CRC patients [56]. In support of this idea, inhibition of Wnt/β-catenin signaling was reported to promote survival of latent metastatic CRC cells [57]. However, given that deficiency for *CFTR* has been reported in a wide range of cancers that arise independently of Wnt/β-catenin signaling, it is likely that CFTR’s function as a tumor suppressor in CRC extends beyond influencing the intestinal stem cell compartment and Wnt/β-catenin signaling. While these additional tumor suppressing mechanisms are not fully understood, from what is known observationally and experimentally about the functions of *CFTR* in normal intestinal tissue and the phenotypes of intestinal tissues that are CFTR-deficient, several potential mechanisms of action are suggested, possibly acting in concert. These mechanisms are described in the following sections. Figure 1 depicts a simple model of CFTR’s influence on the intestinal stem cell compartment and Wnt/β-catenin signaling.

### 2.3. CFTR Deficiency Disrupts Protective Physical Barriers

CFTR influences several inter-related processes in the intestine that are critical for tissue homeostasis. These include the composition of the gut microflora, maintenance of essential barriers protecting the single cell epithelial layer, and homeostasis in the innate and adaptive immune response. Clinical manifestations of CF in the GI tract such as inflammation and obstruction are associated with the dysregulation of these processes, events that also create a favorable landscape for cancer development. In the lumen of the intestinal tract, primarily in the large intestine, trillions of commensal bacteria and other flora such as algae and fungi are found. The microflora, primarily bacteria, are important for nutrient production, such as the production of butyrate, a short chain fatty acid made from bacterial fermentation of dietary fibers. Butyrate is important for colonic health by providing a fuel source in the colon and by its anti-inflammatory action, among a variety of beneficial effects. Microflora also produce signaling molecules that regulate immune cell homeostasis such as lipopolysaccharide (LPS) molecules that can bind to pattern recognition receptors on epithelial cells to activate the intestinal cell immune response. However, it is critically important to prevent contact between microflora and the single cell layer of the intestinal epithelium, principally in the colon. Two primary physical barriers maintain the impenetrability of the colonic epithelium to bacteria. First, the apical surface of the colonic epithelium, facing the crypt and intestinal lumens, is covered by protective dense inner and looser outer mucus layers that are produced by constitutive secretion of mucin 2 (MUC2) mucins from luminal goblet cells. The dense inner mucus layer is largely sterile. The looser outer mucus layer results from partial enzymatic digestion of mucins in the inner layer that migrate out into the outer layer. The outer mucus layer contains an abundant population of commensal bacteria [58]. Secreted MUC2 initially exists in a very condensed form that expands to create the dense inner layer in a process that requires both bicarbonate ions (HCO_3_^−^) and water [59]. The ion channel function of CFTR is directly involved in HCO3- ion efflux into the lumen and indirectly involved in promotion of H_2_O efflux as well [60]. CFTR deficiency, consequently, causes the mucus layers to become dehydrated and dysfunctional. In the distal small intestine of CF infants this causes obstruction, a disorder called meconium ileus [11]. Obstruction also occurs in the proximal large intestine, leading to inflammation and related pathologies in CF patients, a phenotype also observed in *Cftr* KO mice. Further, markedly dilated CFTR-deficient crypts are found to contain an accumulation of unsecreted mucus in the goblet cell population, indicating that deficiency for CFTR may regulate goblet cell secretion as well [61,62]. In addition, the dehydrated dysfunctional mucus layer permits illicit bacterial contact with the epithelia as enlarged bacterial colonies were found in the inner mucus layer of *CFTR* KO ferrets [61]. Than et al. used gene set enrichment analysis (GSEA) [63] to compare gene expression profiles from *Cftr* KO mouse intestine with *Muc2* KO mouse intestine and reported an enrichment in inflammatory gene expression [5], supporting the idea that the loss of *Cftr*, like loss of *Muc2*, permits dysregulated bacterial contact with the epithelial layer. A second protective physical barrier is the tight junctions between the basolateral surfaces of intestinal epithelial cells. CFTR is involved in maintenance of these tight junctions as loss of Cftr in the small intestine of KO mice resulted in enhanced epithelial permeability and disruption of epithelial tight junctions [64]. Several reports indicate that CFTR may mediate tight junction integrity via its C-terminal PDZ-binding domain [64]. This was demonstrated by in vitro studies in airway epithelial cells that reported physical interaction between the CFTR–PDZ binding domain (CFTR–PDZBD) and the PDZ domain of NHERF1 (Na+/H+ exchange regulatory cofactor 1)/ SLC9A3R1 (solute carrier family 9, subfamily A, member 3 regulator), a protein critical for the organization of the actin cytoskeleton and further important for tight junctions [65]. In addition, loss of CFTR could disrupt tight junctions by preventing its protein–protein interaction with TJP1 (tight junction protein 1)/ZO-1 (zona occludins 1), a key protein in both adherens and tight junction assembly and structure [66]. CFTR protein–protein interactions may also mediate tumor suppression by acting as a signaling hub with metastasis suppressor proteins of the NM23 (non-metastatic clone 23)/NDPK (nucleotide diphosphate kinase) family that are proposed to negatively regulate the Ras/PI3K(phosphoinositide 3 kinase) signaling pathway [67]. For example, it was reported that NM23-H1 (NM23-human gene 1) and NM23-H2 (NM23-human gene 2) are present in a complex of proteins near the plasma membrane that bind different domains of CFTR, including NBD1 (nucleotide binding domain 1) [68]. Mutations in CFTR disrupt this complex leading to dysfunction of NM23/NDPK [67].

### 2.4. CFTR Deficiency Causes Dysbiosis

Bacterial dysbiosis is commonly observed in the gut of CF patients and in CF animal models [11,69]. Loss of CFTR causes a dysregulated environment in the intestinal lumen that is associated with disruption of the mucus layer (as described above) and, specifically in CF patients is exacerbated by factors such as nutrient malabsorption, high fat diets, and antibiotic therapy. The degree of dysbiosis is associated with the severity of CF GI disease, GI inflammation, and nutrient uptake deficiency [11]. The first experimental evidence for dysbiosis was bacterial overgrowth observed in the usually-sterile small intestine of *Cftr* KO mice. qRT-PCR of bacterial 16S DNA in flushed mouse small intestine found a 40-fold increase in total bacteria with reduced species diversity [70]. Another report employed phylogenetic microarray analysis of flushed mouse small intestine and found a significant reduction in bacterial richness, evenness, and species diversity. Specifically, there was a reduction in the abundance of protective bacteria such as *Acinetobacter Iwoffii* and *Lactobacilliales* members, but a rise in *Mycobacteria* species and *Bacteriodes fragilis* bacteria implicated in infection and immunomodulation in the GI tract [71]. In following studies, the microbiota composition of CF vs. non-CF individuals was evaluated employing species-specific and metagenomic analysis of 16s rRNA DNA from patient feces. Compared with non-CF controls the microflora in CF patients showed markedly decreased diversity, evidence that CF-related changes in the gut lumen not only permitted enhanced illicit bacterial contact with the epithelia but in addition altered the composition of the microbiota [72,73,74]. Recently, changes in the microbiome of CF patients have been linked to potentially oncogenic functional consequences [75]. Meeker et al. [76] employed germ-free *Cftr* KO mice treated with specified pathogen-free (SPF) fecal transplants and reported that *Cftr* mutations alone were sufficient to alter the fecal GI microbiome, with enrichment in the proportion of *Escherichia/Shigella* (>250-fold) and a depletion of *Lachnoclostridium* and *Parabacteriodes.* This result is consistent with findings in the fecal microbiome of children with CF [77]. The Meeker et al. study further showed that colonized *Cftr* KO mice had an increase in mesenteric lymph node and spleen TH17^+^ cells compared with non-*Cftr* KO mice, evidence that CFTR deficiency altered the adaptive immune response [76]. In addition, CF patients with specific *CFTR* mutations and bacterial dysbiosis are vulnerable for the development of Crohn’s disease [78]. In another recent study in human CF patients Dayama et al. [79] described interactions between the gut microbiome and host gene regulation, resulting in enrichment of oncogenic pathways. They conducted RNA-Seq analysis of colonic mucosa samples from CF patients and healthy controls as well as 16S rRNA sequencing to characterize the colonic microbiome. They found more than 1500 genes differentially expressed in CF patients, with enrichment for genes related to CRC, including CRC metastasis, tumor suppression, P53 (tumor protein 53), and the mTOR (mammalian target of rapamycin) pathway. Further, CF patients showed decreased microbial diversity, decreased abundance of butyrate producing bacteria, such as *Ruminococcaceae* and *Butyricimonas*, and an increased abundance of taxa such as *Actinobacteria* and *Clostridium*. Butyrate, a product of bacterial fermentation by *Ruminococcaceae* and *Butyricimonas* (and other bacteria) of dietary fibers, is considered to prevent CRC via epigenetic regulation of gene expression [80,81] while *Actinobacteria* and *Clostridium* are among the taxa associated with CRC development and progression [82,83]. An integrative analysis by Dayama et al. identified CRC-related genes, including *lipocalin 2* (*LCN2*) and *dual oxidase 2* (*DUOX2*), which were correlated with the abundance of CRC-associated bacteria such as *Veillonella* [79]. These findings are consistent with more general findings showing increased evidence of an association between alterations in the intestinal microbiome with CRC [75,84]. For example, a recent study by Hans Clevers and co-workers reported that a colibactin-induced genotoxic mutational signature caused by *pks*^+^ *E. coli* was identified in a subset of more than 3000 metastatic CRC patients [85]. 

### 2.5. Loss of CFTR Promotes Immune Infiltration of the Epithelial Layer and Lamina Propria

Disruption of physical barriers in the intestine permits illicit access of bacteria to resident immune cells of the lamina propria as well as infiltration of the epithelia by immune cells. While immune cell infiltration and inflammation can lead to damage in the epithelia, only a single study thus far has observed this type of damage in CF patients, duodenal lesions detected by capsule endoscopy [86]. However, infiltration of immune cells has been observed in the intestine of both CF patients and in mouse CF models but this infiltration did not cause apparent morphological and histological damage [87]. Immune cell infiltration in CF patients was found following whole gut lavage [88], and assays to detect fecal calprotectin [86,89] and rectal nitric oxide [90]. In *Cftr* KO mice, gene expression analysis in mouse small intestine demonstrated upregulation of genes expressed by granulocytes. This finding was confirmed by microscopic analysis that detected elevated numbers of mast cells and neutrophils in the same tissue [87]. In support, Liu et al. found immunocytochemical evidence of elevated numbers of macrophages and neutrophils in the small intestine of *Cftr* F508del mice [53]. There is also emerging evidence that CF patients demonstrate dysregulation of CFTR expression in various immune cell populations that contribute to aberrant immune cell activity in several organs, in particular the lung, but also likely in the GI tract. CFTR expression and dysregulation has been detected in monocytes/macrophages and dendritic cells of the peripheral innate immune response. These cells can also influence adaptive T cell responses via antigen presentation. CFTR may also influence the immune response through its expression in lymphocytes and NK cells [91,92]. Furthermore, CFTR dysregulation of neutrophils in CF patients has been shown to cause neutrophil intrinsic impairment linked to degranulation [93].

### 2.6. Loss of CFTR Promotes Pro-Inflammatory Signaling

Loss of CFTR has been associated with activation of NF-κB signaling pathways, primarily in in vitro models. Xie et al. and Zhang et al. studied cell lines generated from several different types of cancer. They reported that loss of CFTR led to NF-κB activation, upregulation of UPA (urokinase-type plasminogen activator) expression, and increased cellular migration and invasion [94,95]. Studies in *CFTR-*deficient Caco-2, HT-29, and HEK293 human CRC cells reported elevated levels of pro-inflammatory cytokines. Specifically, there was an increase in the expression of NF-κB pathway members, including an elevation in TNF-α (tumor necrosis factor alpha), IL-6 (interleukin-6), and IL-1β (interleukin-1 beta)-induced secretion of IL-8 (interleukin-8), COX-2 (cyclooxygenase-2), and PGE_2_ (prostaglandin E2)_,_ along with enhanced activities of ERK1/2 (extracellular signal-related kinases 1/2), MAPK (mitogen-activated protein kinase), IκBα (NF-kappa-B inhibitor alpha), and NF-κB [96,97,98,99]. Massip-Copiz et al. reported that loss of CFTR caused NF-κB activation following autocrine signaling by IL-1β [100]. Furthermore, loss of CFTR was reported to cause downregulation of the hedgehog signaling pathway, an effect linked to heightened inflammatory signaling [101]. Finally, dysregulation of calcium homeostasis has been reported in CF bronchial epithelium both as a result of chronic inflammation [102], and as a direct result of mislocalized F508del-CFTR [103,104,105]. The increased TRPC6 (transient receptor potential cation subfamily C, member 6) Ca^2+^ entry, increased store-operated Ca2^+^ entry (SOCE), and aberrant mitochondrial buffering capacity seen in CF cells is likely altering the inflammatory response as well as various other pathways utilizing Ca^2+^ as a second messenger. While evidence for this influence of dysregulated Ca^2+^ signaling in CFTR-deficiency mediated inflammatory signaling in CRC is thus far weak, it remains a research area for heightened investigation. 

Dysregulation of barrier integrity, dysbiosis, immune cell infiltration, and inflammation work together in disruption of colonic homeostasis, altering the landscape to favor CRC development.

**Figure 2:**
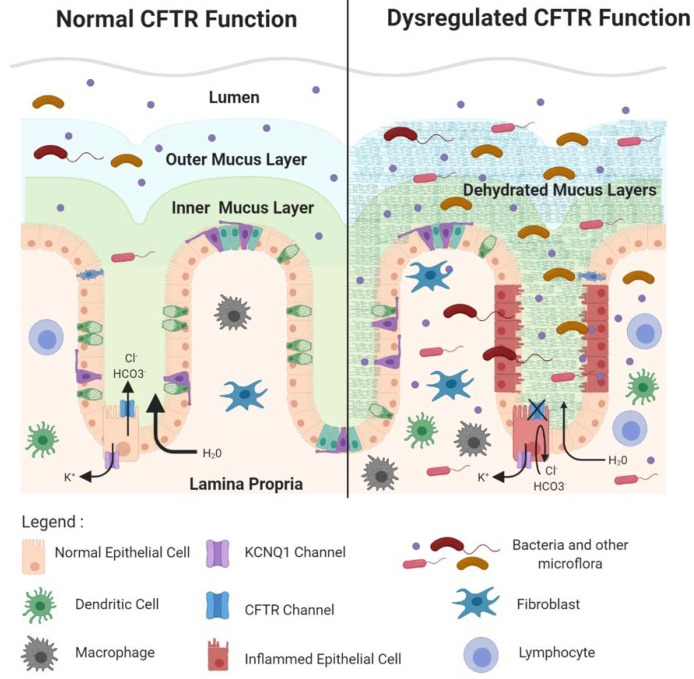
CFTR deficiency disrupts protective physical barriers and leads to dysbiosis. CFTR deficiency causes a failure of intestinal cell chloride and bicarbonate ion efflux and accompanying water efflux. This results in dehydration of the mucus layer, making it permissive to bacterial passage, and also causing intestinal obstruction. Disruption of the epithelial barrier leads to infiltration of commensal and pathogenic bacteria, inflammation, epithelial tissue damage, and immune cell infiltration. These alterations in the intestinal landscape (mutations, inflammatory signaling) create favorable conditions for CRC initiation and progression. Figure created using BioRender.com.

Dysregulation of physical barrier integrity permits illicit bacterial contact with the intestinal epithelia, further attracting cells of the innate and adaptive immune system that can promote both direct and indirect pro-inflammatory responses [106,107,108,109]. For example, shedding of lipopolysaccharides that comprise the outer membrane of Gram-negative bacteria, can directly promote the production of pro-inflammatory cytokines via their interactions with toll-like receptors (TLR) on the intestinal epithelia [110], while, indirectly, illicit bacterial contact can activate cytokine signaling to recruit lymphocytes. There is evidence that CFTR is involved in mediating some of these direct and indirect effects. In studies employing primary biliary epithelial cells, Strazzabosco and colleagues reported binding of CFTR to inhibitors of Src (SRC proto-oncogene, non-receptor tyrosine kinase) that promoted their physical interactions with Src to inhibit its activity. Knockout of *CFTR* in these cells caused these Src inhibitors to be mislocalized, resulting in Src activation. Src activation then synergized with bacterial products such as LPS causing activation of NF-κB signaling and eventually disruption of both adherens and tight junctions [111]. Inflammatory responses are also associated with dysregulation of intestinal stem cell homeostasis, causing enhanced proliferation and expansion of the stem cell compartment via reversion of differentiated early transit amplifying cells back to stem cells [112]. Disruption of physical barriers caused by illicit bacterial contact can also lead to altered migration and invasion of cancer cells [113]. Dysbiosis resulting from loss of CFTR may promote the production of bacterial taxa that are prevalent in colorectal cancer. For example, studies in *Cftr* KO mice found an increase in *Bacteroides fragilis* [71], a species of bacteria associated with CRC because of its link with Stat3 (signal transducer and activator of transcription 3) signaling via a Th17+ (T helper cell 17) immune response [114]. See Figure 2.

### 2.7. CFTR Is Associated with Altered Stress Responses

**Figure 3:**
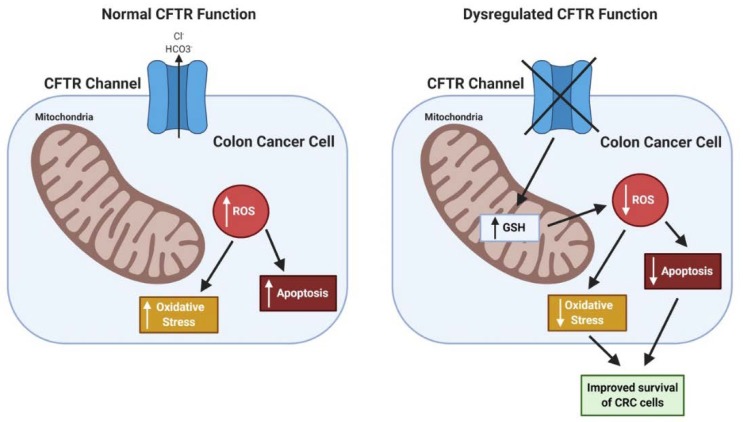
CFTR deficiency improves survival of CRC cells under oxidative stress. Cancer cells are subject to oxidative stress via increased production of reactive oxygen species (ROS) and are therefore susceptible to oxidative-stress-induced cell death. A potentially protective mechanism for CRC cells involves CFTR-deficiency leading to increased retention of the antioxidant glutathione (GSH) in mitochondria. Enhanced GSH could cause decreased ROS levels, decreased oxidative stress and decreased apoptosis. Through this mechanism CFTR-deficiency may promote survival of CRC cells. Figure created using BioRender.com.

In the literature, loss of *CFTR* is associated with both a rise in cellular oxidative stress caused by dysfunction of mitochondria [97,115,116,117] and a reduction in cellular oxidative stress by retention in the cells of the antioxidant glutathione (GSH) [116]. Loss of CFTR in human Caco-2/15 CRC cells resulted in an elevation of lipid peroxidation along with a reduction in anti-oxidant factors such as the enzymes glutathione peroxidase and catalase [118]. Further, CFTR-deficiency has been linked to the downregulation of cellular autophagy caused by transglutaminase-2 (TG2) activation. TG2 cross-linking activity results in key autophagy proteins being bound up in aggresomes [119]. In addition, there is evidence that CFTR expression (mRNA, protein) and function can be repressed by HIF-1 in hypoxic epithelium, which is common in CRC, and most epithelial cancers [120]. How these various alterations in stress responses influence cancer development, particularly in the context of loss of CFTR, remains unclear. Overall, the potential impact of CFTR deficiency in CRC on oxidative stress may be context dependent, as may be true for cancers in general. Because of high rates of metabolism in cancer cells, oxidative stress and accompanying production of reactive oxygen species (ROS) is increased. While increased ROS can promote cancer development via mechanisms such as DNA damage and mutations, ROS is also harmful to both normal and cancer cells with excess DNA damage leading to apoptosis. One potential hypothesis is that CFTR deficiency protects CRC cells from ROS-induced cell death via retention of antioxidants such as GSH and other mechanisms. Preliminary studies in our research group employing CFTR-deficient human Caco-2 CRC cells indicate that CFTR-deficiency promotes the survival of CRC cells after treatment with oxidative-stress-inducing agents such as menadione [121]. Similar results have been observed in *Cftr*-deficient mouse colon organoids [122]. See Figure 3.

### 2.8. The Role of CFTR in Non-Colonic Cancers

*CFTR* is widely expressed in epithelial tissues, and as discussed above, CFTR influences epithelial cell homeostasis via a range of mechanisms, therefore it was not unexpected that *CFTR* dysregulation has been reported in a wide range of epithelial cancers. Upregulation of *CFTR* expression has been associated with several cancers such as gastric and ovarian cancers [123,124,125]. But for the great majority of cancer studies, loss of *CFTR* expression and/or activity is linked with cancer incidence, and in the majority of these reports, *CFTR* mutations or downregulation of CFTR expression in epithelial cancers is associated with rapid cancer growth, epithelial to mesenchymal transition (EMT), a reduction in cellular apoptosis, enhanced metabolic potential, and elevated patient morbidity and mortality. Down regulation of *CFTR* expression and/or mutations that disrupt CFTR function have been reported in non-small-cell lung cancer (NSCLC) [33,86,126,127], glioblastoma [128], bladder cancer [34,35,36], esophageal cancer [129,130], pancreatic cancer [131,132,133], nasopharyngeal cancer [134], prostate cancer [94], and breast cancer [37,95]. In many of these malignancies, loss of *CFTR* correlated with increased tumor stage and reduced disease-free or overall survival. Inherited heterozygous *CFTR* mutations have been associated with an increased risk of pancreatic cancer among both younger [131,132,133] and older patients [4]. Yet, a reduction in *CFTR* mRNA expression in most non-colonic cancers studied has not been linked to germline mutations, although germline mutations were not likely tested for in most cases. CF-related germline mutations are not the likely cause of reduced CFTR mRNA expression in these cancers as F508del, which represents ~ 70% of CF alleles, results in a 90% decrease in protein levels but a very modest or no reduction in mRNA [135]. Reasons for a loss of *CFTR* mRNA expression include promoter hypermethylation as has been reported in lung, breast, head and neck, and bladder cancer studies [34,35,36,37,38] and somatic *CFTR* mutations as seen in NSCLC [32]. Furthermore, cigarette smoke (CS) was reported to downregulate *CFTR* mRNA expression and downregulation of CFTR expression has been associated with the development of chronic obstructive pulmonary disease (COPD) [136]. A link between CS, downregulation of CFTR, and lung cancer was reported in a study that correlated CS and *CFTR* downregulation with enhanced oncogenesis in A549 lung cancer cells [137]. 

### 2.9. CFTR Therapies: Clinical Implications in CRC

CF therapies to correct the underlying defects caused by specific *CFTR* mutations are now in the clinic. For example, potentiator drugs can enhance anion efflux via CFTR channels already situated on the plasma membrane and corrector drugs can cause correct folding of mutant CFTR. Modulators such as ivacaftor, elexacaftor, tezacaftor, and lumacaftor are now in use to treat CF [138]. Ivacaftor is a potentiator employed in treatment of >25 CF-causing mutations that disrupt CFTR gating as well as residual function, and *CFTR* splice mutations. Tezacaftor, elexacaftor, and lumacaftor are correctors that can enhance the function of the F508delta mutation that represents ~ 70% of CF alleles. Most recently, a combination of three small molecules (TRIKAFTA) comprising two correctors (tezacaftor and elexacaftor) plus one potentiator (ivacaftor) have been approved by the FDA for patients who carry one or two copies of the F508del allele [139,140]. With the development of TRIKAFTA, modulator therapies are available to treat 90% of CF patients. Notably, these therapeutic drugs are now targeting CF-related gastrointestinal disorders, with the possibility of future application to cancer [141]. For example, CF patients with G155D mutations are now being treated with Ivacaftor, resulting in improvement for pancreatitis [142]; and of pH in the proximal small intestine, an improved clinical phenotype associated with increased bicarbonate secretion; increased cell motility; and overall improvement in clinical outcomes [143]. Lumacaftor was also demonstrated to improve bicarbonate permeability in CF patients with F508del mutations [144]. Ivacaftor treatment beneficially improved colonic microbiota, such as an increase in *Akkermansia* and a reduction in *Enterobacteriaceae*. Ivacaftor treatment also caused a major decrease in GI inflammation in CF patients [145]. Other drugs, either singly or in combination are being developed. For example, the combination of ivacaftor with 5-nitro-2-(3-phenylpropylamino) benzoate, has been shown to synergize in CF patients with G551D mutations [146]. Notably, these targeted therapies may be CRC preventative for the 3%–4% of Caucasians who carry germline *CFTR*-inactivating mutations. These individuals are at an enhanced risk for developing CRC [4], either via *CFTR* haploinsufficiency or LOH (loss of heterozygosity). Beyond US FDA-approved drugs, novel modulators known as amplifiers, such as PTI-428, are in current clinical trials. PTI-428 is proposed to increase the translation of *CFTR* mRNA and thus complement the action of corrector drugs [11]. PTI-428 may also be useful for augmenting CFTR activity in sporadic CRC patients with low CFTR protein expression. Testing protocols currently employed for screening and diagnosis of CF could be repurposed for adjusting the timing of early detection assays (such as colonoscopy screening), and even for the prevention of CRC. Genetic testing for the panel of >99% of CF-causing mutations is made available to all pregnant couples. This testing could also be adaptable for early detection of CRC in CF carriers. A major technical breakthrough to evaluate new CF therapies has been the development of patient-derived colorectal organoids [147]. Evidence for their applicability has been demonstrated in rectal organoids developed from CF patients that have been used to evaluate several CF drugs, including ivacaftor and lumacaftor [148].

## 3. Conclusions

Loss of *CFTR* causes disruption to a wide range of cellular processes that can promote carcinogenesis but it still remains unclear which of these dysregulated processes, alone or together, are most critical for CRC development and progression in the context of *CFTR*-deficiency. One area that cancer investigators might focus on is the influence of CFTR on the intestinal stem cell (ISC) compartment as ISCs are the source of putative CRC progenitor cells [23]. ISCs are located at the crypt base in the colon, the location of peak *CFTR* expression. To protect the vital long-lived ISCs from a hostile luminal environment the crypt contains several key protective mechanisms. These include a unique microflora, especially in the inner mucus layer, with a reduction in the quantity and diversity of bacteria compared with the outer intestinal lumen [149]. The dense inner mucus layer prevents illicit contact between bacteria and bacterial products and the single cell intestinal epithelial layer of the crypt. Sensors participate in this protective mechanism. When bacterial products such as LPS are detected by goblet cells at the crypt neck additional protective mucins are released [150]. Mitigation of harmful oxidative stress is another evolved protective mechanism in the crypt. For example, ISC survival is mediated by physical interactions between the cytosolic innate immune sensor Nod2 (nucleotide-binding oligomerization domain-containing protein 2) and the bacterial peptidoglycan motif MDP (muramyl dipeptide) [151]. These mechanisms are particularly important to block inflammation that can jeopardize crypt homeostasis and promote oncogenesis. For example, NF-κB has been demonstrated to synergize with Wnt/β-catenin signaling in crypt cells to cause reversion of early differentiated crypt cells to stem-like cells with the ability to cause tumorigenesis [112]. All of these observations emphasize the priority of investigating how loss of CFTR impacts on the crypt and ISCs, studies that can be especially informative by employing crypt-derived organoid models. Finally, repurposing of drugs to treat GI complications in CF patients have now reached the clinic. While these mutation-specific targeted CF drugs have not yet reached the cancer clinic, they could be used in the clinic in the near future, along with the re-purposing of other ion channel activators and blockers as more becomes known about the precise contribution of ion channels to cancer development.
